# Study on Current Levels of Physical Activity and Sedentary Behavior among Middle School Students in Beijing, China

**DOI:** 10.1371/journal.pone.0133544

**Published:** 2015-07-16

**Authors:** Jiali Duan, Huanhuan Hu, Guan Wang, Takashi Arao

**Affiliations:** 1 Institute of School Health, Beijing Center for Diseases Control and Prevention, Beijing, China; 2 Lab of Exercise Epidemiology, School of Sport Sciences, Waseda University, Saitama, Japan; 3 Lab of Exercise Epidemiology, Faculty of Sport Sciences, Waseda University, Saitama, Japan; The Ohio State University, UNITED STATES

## Abstract

**Introduction:**

This study aimed to determine current levels of physical activity and sedentary behavior in middle school students on the basis of grade, sex, student attitudes toward physical education, and residence location.

**Methods:**

In 2013, a cross-sectional study of 1793 students aged 12 to 15 years was conducted across eight middle schools in Beijing, China. Four schools were selected from an urban district and another four schools were from a suburban district. Physical activity and sedentary behavior data were collected using the commonly used school-based Chinese version of the China Health and Nutrition Survey.

**Results:**

The mean age of sampled students was 13.3±1.0 years; 51.5% were boys. Approximately 76.6% of students reported having three 45-minute physical education classes every week. A total of 35.6% students spent ≥1 h/day performing moderate to vigorous physical activity (MVPA) during school, and 34.9% spent ≥1 h/day in MVPA outside school time. Approximately half (49.7%) of the students engaged in reading, writing, or drawing for ≥2 h/day, and 42.9% reported screen time for ≥2 h/day. Although boys spent more time engaged in physical activity than girls did, they also spent more time exhibiting sedentary behavior. Each 10-unit increase in attitudes toward physical education was associated with an increased odds of 1.15 (95%CI: 1.09–1.20) for spending more than 1 h/day on MVPA. Students in suburban schools reported engaging in physical activity less when compared with those in urban schools.

**Conclusion:**

The majority of our students did not meet the current physical activity recommendations, and about half of the students spent excessive time engaging in sedentary behaviors. Findings from this study highlight a positive association between student attitudes toward physical education and physical activity. Studies are needed to further explore the role of student attitudes toward physical education in promoting physical activity among Chinese students.

## Introduction

It is widely accepted that engaging in regular physical activity is positively associated with a range of beneficial childhood health and fitness outcomes [1, 2]. A recent review suggested that aerobic physical activity was positively associated with children’s cognition, academic achievement, and psychosocial function [3]. However, despite such benefits, international data reports that about 80% of adolescents (13–15 years old) spend less than 60 min of moderate to vigorous physical activity (MVPA) per day [4]. Previous research suggested a low level of physical activity among Chinese students [5,6,7]. A study conducted in four cities in China indicated that only 12.2% of students met the current physical activity recommendations of 60 min of MVPA per day [8]. A study shows that the prevalence of spending more than 2 hours per day on screen time increased from approximately 10% in 1997 to approximately 40% in 2006 in Chinese children [9].

There is increasing evidence that youth physical activity can be influenced by demographic, psychological, social, environmental, and other factors [10,11]. Studies show that boys are more active than are girls [4,12]. Emerging evidence has shown that students who show a more positive attitude toward physical education are more likely to participate in physical activity outside of school [13,14]. Research on physical activity among urban and rural adolescents has produced equivocal findings. Some studies reported that urban adolescents were more physically active than rural youths [15,16]. However, some conflicting evidence also exists [17,18]. Further research on geographical differences in physical activity in students is needed.

Mounting evidence suggests that China is facing an increase in the prevalence of obesity, hypertension, and metabolic syndromes among school-aged children [19,20,21]. Decreased physical activity is touted as a probable cause of a decline in pediatric health and fitness levels [22,23]. To promote physical activity among students, the Chinese government has issued a series of mandatory guidelines since 2007 [23]. These guidelines require middle and high schools to provide at least two physical education classes per week, including an additional 60 minutes of daily exercise after class. However, there remains a paucity of data regarding physical activity among Chinese youths since publication of these national guidelines.

In this study, we sought to update the information regarding physical activity levels among middle school students, using data from a cross-sectional survey in Beijing, China. The main objective was to determine current physical activity and sedentary behavior levels among middle school students, although we also sought to explore disparities in contributing factors such as grade, sex, student attitudes toward physical education, and residence location.

## Methods

The ethical review boards at the Beijing Center for Disease Control and Prevention and Waseda University granted us permission to conduct this study. Written informed consent was obtained from the principals, teachers, students, and their custodians prior to data collection. Before instrument administration, students were informed that their participation in the survey was voluntary. The questionnaire was completed anonymously by students.

### Study design and participants

A cross-sectional study was conducted in eight state middle schools in Beijing. Students from 48 classes (grades 7, 8, and 9) were invited to take part in the survey.

### Sampling

Beijing Municipality consists of six urban districts, eight suburban districts, and two rural counties, all of which were classified according to their location and economic development. In total, there were 630 state middle schools. Middle school students are from 13 to 15 years of age. Participants for this study were students from eight state middle schools in two districts. Initially, two districts were contacted to take part in this study (an urban district (43 state middle schools) and a suburban district (47 state middle schools)), from which four middle schools were subsequently contacted to participate. Our sample was not randomized, as we contacted schools directly to recruit participants. In each school, there were four classes per grade on average. Two classes in each grade level were contacted to participate in this survey, and the students in selected classes were invited to fill out the adapted school-based Chinese version of the China Health and Nutrition Survey and the Chinese version of the Students’ Attitudes toward Physical Education Scale (SPEA) [7,24].

### Measurements

Physical activity data were collected using the adapted school-based Chinese version of the China Health and Nutrition Survey, which has been conducted since 1989 [7]. Survey questions were used to identify the physical activities inside and outside school (sports/exercise) and sedentary activities outside of school (reading/writing/drawing, using computer, watching TV/videos, or sitting games) in which students were regularly engaged. For all activities identified, respondents were queried regarding the frequency and average duration (e.g., h/day and min/day) of involvement. Sport and exercise were categorized as moderate to vigorous activity (MVPA), while sedentary activities were considered light, in accordance with previous reports [7,25].

A validated Chinese version of the SPEA was used to measure student attitudes toward physical education [24]. The SPEA consists of 20 items. Response choices were on a 5-point Likert response scale ranging from “strongly disagree” (1) to “strongly agree” (5), with a total possible score of 100 points for overall attitude. A higher score indicates a more positive attitude.

### Procedures

The principals of selected schools were informed of the study objectives and procedures, at which point, they introduced the study team to the school nurses, physical education teachers, and students. Before beginning data collection, all school nurses received a 45-minute training session provided by the principal investigator. The training focused on familiarizing interviewers with the study protocols and questionnaires. This also included how to respond if a student needed assistance and how to address any queries on meaning or interpretation of questions. The questionnaires was completed in the classroom in the presence of school nurses.

### Data management and statistical analysis

Data were double entered and crosschecked using Epi Info version 6 statistical software. In accordance with a previous report [5], weekly minutes of each type of sedentary activity and outside-school MVPA were truncated to 1680 (4 h/day), while weekly minutes of in-school MVPA were truncated to 600 (2 h/day). To be comparable to previous reports [5,7], we established a sample proportion that met the current physical activity recommendation for youth [23], which was MVPA in or outside school for ≥ 1 h/day. Sedentary activity classification (≥2 h/day) and screen time (computer use and television or video watching and video game of ≥2 h/day) that exceeded the international recommendation [26] were also established.

Descriptive statistics were generated for reporting any defined activity performed by the sample population, as well as the median and interquartile range of time distribution (min/week) for each activity [5,7]. School grade and sex group differences for the proportion of students who engaged in MVPA ≥ 1 h/day or spent ≥ 2 h/day in sedentary behaviors, were analyzed using Chi-square tests. Wilcoxon-Mann-Whitney tests and Kruskal-Wallis tests were used to test time differences in activity classifications.

Associations between sex, grade, residence location, student attitudes toward physical education, and MVPA and screen time were analyzed. Survey logistic regression analysis was applied to account for school-level intraclass correlation. Stepwise elimination process (entry criterion, 0.10; stay criterion, 0.05) was applied for model choice. All statistical analyses were performed using SAS version 9.3 (SAS Institute, Inc., Cary, North Carolina), where P-values of <0.05 were consider significant.

## Results

Of 1793 invited students, 1715 (95.6%) completed the questionnaires. The mean age was 13.3±1.0 years, 51.5% (883) were boys, and 47.9% (821) were from suburban schools.

### Physical activity

Approximately 5.1% of sampled students reported having one physical education class every week, 1.5% reported having two 45-minute physical education classes every week, 76.6% reported having three physical education classes every week, and 16.7% reported having four or five physical education classes every week. Overall, the mean of the attitude toward physical education was 75.5±13.4 out of 100.

A total of 35.6% students spent ≥1 h/day in MVPA during school, and 34.9% spent ≥1 h/day in MVPA outside of school time (Tables 1 and 2). Significant school differences, sex differences, and school location differences were found for these physical activities.

**Table 1:** Prevalence of physical activity and sedentary activity in Beijing school students by school.

	School 1	School 2	School 3	School 4	School 5	School 6	School 7	School 8	*P*
**MVPA %**									
Prevalence of MVPA in school ≥1 h/day	36.6	38.5	36.7	42.2	32.7	38.3	19.0	41.8	***
Prevalence of MVPA outside school ≥1 h/day	28.6	37.6	39.4	40.8	31.7	33.5	33.7	41.0	
Prevalence of total MVPA ≥1 h/day ^a^	56.3	55.3	54.0	69.3	47.6	52.7	38.7	61.6	***
**Sedentary activity n,%**									
Prevalence of reading/writing/drawing ≥2 h/day	57.1	57.5	36.7	40.8	61.1	41.5	49.8	53.4	***
Prevalence of computer use ≥2 h/day	20.1	22.1	27.4	22.5	23.1	28.2	17.6	22.6	
Prevalence of TV/video watching ≥2 h/day	4.0	8.0	9.3	10.1	5.8	16.0	5.0	11.0	***
Prevalence of screen time ≥2 h/day ^b^	39.7	35.8	46.9	40.4	46.6	53.7	35.1	52.1	***
Prevalence of sitting games ≥2 h/day	0.5	3.1	4.0	2.3	2.4	4.8	1.4	3.4	

Abbreviations: MVPA, moderate to vigorous physical activity; TV, television.

^a^Total MVPA: the total MVPA in and outside of school

^b^ Screen time: computer use + TV/video watching + video games

*P < 0.05

** *P* < 0.01

*** *P* < 0.001

**Table 2:** Prevalence of physical activity and sedentary activity in Beijing school students by gender and school location.

	Gender		Location		
	Boys (n = 883)	Girls (n = 821)	Urban (n = 894)	Suburban (n = 821)	Overall (n = 1715) ^a^
**MVPA n,%**					
Prevalence of MVPA in school ≥1 h/day	333 (37.7)	274 (33.4)	327 (36.6)	283 (34.5)	610 (35.6)
Prevalence of MVPA outside school ≥1 h/day	356 (40.3)	237 (28.9)***	344 (38.5)	254 (30.9)**	598 (34.9)
Prevalence of total MVPA ≥1 h/day ^b^	504 (57.1)	411 (50.1)**	524 (58.6)	396 (48.2)***	920 (53.6)
**Sedentary activity n,%**					
Prevalence of reading/writing/drawing ≥2 h/day	380 (43.0)	464 (56.5)***	430 (48.1)	422 (51.4)	852 (49.7)
Prevalence of computer use ≥2 h/day	239 (27.1)	148 (18.0)***	206 (23.0)	183 (22.3)	389 (22.7)
Prevalence of TV/video watching ≥2 h/day	89 (10.1)	53 (6.5)**	70 (7.8)	72 (8.8)	142 (8.3)
Prevalence of screen time ≥2 h/day ^c^	434 (49.2)	297 (36.2)***	364 (40.7)	372 (45.3)	736 (42.9)
Prevalence of sitting games ≥2 h/day	38 (4.3)	6 (0.7)***	22 (2.5)	23 (2.8)	45 (2.6)

Abbreviations: MVPA, moderate to vigorous physical activity; TV, television.

^a^ 11 students have missing values on gender

^b^ Total MVPA: the total MVPA in and outside of school

^c^ Screen time: computer use + TV/video watching + video games

*P < 0.05

** *P* < 0.01

*** *P* < 0.001

Table 3 presents the proportion of students who engaged in MVPA and the time expended (min/week) during and outside school time according to sex and grade. Girls in grade 7 reported a higher proportion of MVPA outside school compared to girls in grades 8 and 9 (*P* < 0.05). There was no significant difference in the proportion reporting any MVPA during school or outside of school between sexes within grade groups. However, girls engaged in shorter durations of MVPA during school and outside of school when compared to boys.

**Table 3:** MVPA activities in Beijing school students by gender and grade.

	Grade	% reporting > 0 min ^a^		Median (IQR) (min/week)	
		Boys	Girls	Boys	Girls
During school MVPA	7	209/297 (70.4)	229/300 (76.3)	300 (180–500)	225^c^ ** (150–450)
	8	219/303 (72.3)	214/274 (78.1)	300 (150–600)	225^c^ * (150–450)
	9	198/283 (70.0)	183/247 (74.1)	250 (150–450)	250^c^ * (175–450)
	Total	626/883 (70.9)	626/821 (76.2)^a^ *	300 (150–525)	237^a^ ** (150–450)
Outside school MVPA	7	209/297 (70.4)	215/300 (71.7)^b^ *	450 (240–855)	330^c^ ** (185–660)
	8	215/303 (71.0)	178/274 (65.0)	480 (270–860)	360^c^ ** (240–690)
	9	196/283 (69.3)	154/247 (66.6)	490 (270–865)	330^c^ *** (210–620)
	Total	620/883 (70.2)	547/821 (66.6)	480 (270–857)	345^a^ *** (210–650)

Abbreviations: MVPA, moderate to vigorous physical activity; IQR = interquartile range, computed for only those reporting any (>0 min).

^a^ Descriptive statistics were calculated for total reported time in MVPA (sum of in-school, and outside school). Descriptive statistics herein are therefore presented as the proportion reporting any defined MVPA and the median and interquartile range (IQR) of distribution of time (min/week) for those reporting any of MVPA. ^a^ gender difference regardless of grade

^b^ grade difference within gender

^c^ gender difference within grade.

* *P*<0.05

** *P*<0.01

*** *P*<0.001

### Sedentary activity

Approximately half (49.7%) of the students engaged in reading, writing, or drawing for ≥2 h/day, and 42.9% of the students reported engaging in screen time for ≥2 h/day. Relatively few students (2.6%) engage in sitting games for ≥2 h/day, which included playing video games. For screen behaviors, 22.7% of students use the computer for ≥2 h/day, while even fewer students reported watching television or videos ≥2 h/day (8.3%) (Tables 1 and 2). Significant school differences and sex differences were found for the above mentioned sedentary activities.

Table 4 presents the proportion of students who engaged in any sedentary activity and the time (min/week) expended outside school according to sex and grade. Significant grade group differences were found for the proportion reporting any time spent reading, writing, and drawing, and cumulative weekly time spent reading, writing, and drawing, using a computer, or watching television or videos, or participating in sitting games. There was a significant difference in the reporting time spent on sedentary activities between sexes within grade groups. Girls reported that they cumulatively spent more time reading, writing, and drawing per week than boys. In contrast to girls, boys spent more time using the computer, watching television or videos, or engaging in sitting games.

**Table 4:** Sedentary activities outside school in Beijing school students by gender and grade.

	Grade	% reporting > 0 min		Median (IQR) (min/week)	
		Boys	Girls	Boys	Girls
Reading/writing/drawing	7	282/297 (94.9)	294/300^c^ * (98.0)	645^a^ * (420–930)	790^a^ ^c^ * (490–1110)
	8	285/303 (94.1)	268/274^c^ * (97.8)	840 (510–1120)	930^c^ * (595–1240)
	9	251/283 (88.7)	235/247^c^ *** (95.1)	960 (590–1330)	1080^c^ * (780–1380)
	Total	818/883 (92.6)	797/821^d^ *** (97.1)	780 (480–1130)	925^d^ *** (580–1240)
Computer use	7	244/297 (82.2)	237/300 (79.0)	480^a^ * (230–760)	380^a^ ^c^ * (170–670)
	8	251/303 (82.8)	240/274 (87.6)	700 (360–1200)	485^c^ *** (300–920)
	9	232/283 (82.0)	212/247 (85.8)	630 (365–1080)	480^c^ *** (240–715)
	Total	727/883 (82.3)	689/821 (83.9)	555 (300–1055)	450^d^ * (240–770)
TV/video watching	7	234/297 (78.8)	239/300 (79.7)	270^a^ * (160–430)	270 (140–420)
	8	239/303 (78.9)	220/274 (80.3)	390 (220–600)	270^c^ ** (170–510)
	9	207/283 (73.1)	191/247 (77.3)	320 (210–630)	270^c^ ** (180–420)
	Total	680/883 (77.0)	650/821 (79.2)	320 (200–540)	320 (160–450)
Sitting games	7	130/297 (43.8)	93/300^c^ ** (31.0)	123^a^ * (70–300)	140 (60–290)
	8	156/303 (51.5)	81/274^c^ *** (29.6)	210 (120–420)	150^c^ ** (80–270)
	9	126/283 (44.5)	64/247^c^ *** (25.9)	240 (120–480)	120^c^ *** (60–270)
	Total	412/883 (46.7)	238/821^a^ *** (29.0)	210 (110–420)	140^d^ ** (60–270)

Abbreviations: IQR = interquartile range, computed for only those reporting any (>0 min); TV, television.

^a^ grade difference within gender

^b^ grade difference regardless of urban/suburban residence

^c^ gender difference within grade

^d^ gender difference regardless of grade.

* *P*<0.05

** *P*<0.01

*** *P*<0.001

### Correlates of screen time and MVPA

Table 5 presents the correlates of spending more than 2 hours per day in screen time and spending more than 1 hour per day in MVPA, respectively. Boys were 1.70 times (OR: 1.70, 95%CI: 1.33–2.16) more likely to exceed the 2 hour per day screen time limit compared with girls. Students in grade 8 and 9 were more likely (Grade 8, OR: 1.88, 95%CI: 1.67–2.12; Grade 9, OR: 1.45, 95%CI: 1.02–2.04) to spend more than 2 hours per day on screen behaviors compared with those at grade 7. Boys were 37% more likely (OR: 1.37, 95%CI: 1.10–1.69) to spend more than 1 hour per day on MVPA compared with girls. Compared to the students who lived at suburban areas, students in urban areas were 1.59 times (OR: 1.59, 95%CI: 1.03–2.47) more likely to spend more than 1 hour per day on MVPA. Each 10-unit increase in attitudes toward physical education was associated with an increased odds of 1.15 (OR: 1.15, 95%CI: 1.09–1.20) for spending more than 1 hour per day on MVPA.

**Table 5:** Correlates of spending more than 2 hours/day of screen time outside school and spending more than 1 hour/day of MVPA in and outside school^a^.

	Screen time ^b^		MVPA	
	Screen time ≥2 h/day	Adjusted OR	MVPA ≥1 h/day ^c^	Adjusted OR
Sex (%)				
Girls	36.2	1	50.1	1
Boys	49.2	1.70 (1.33–2.16)	57.1	1.37 (1.10–1.69)
Grade (%)				
7	35.1	1	54.2	
8	50.2	1.88 (1.67–2.12)	54.2	
9	43.8	1.45 (1.02–2.04)	52.4	Not included in final model
Attitude scores ^d^ mean (SD)	75.5 (13.6)	Not included in final model	77.5 (12.7)	1.15 (1.09–1.20)
Residence (%)				
Suburban	45.3		48.2	1
Urban	40.7	Not included in final model	58.6	1.59 (1.03–2.47)

Abbreviations: MVPA, moderate to vigorous physical activity.

^a^ Stepwise backward elimination SURVEYLOGISTCI regression was used to model the correlates initially from a full model with all variables in the left column in the Table

^b^ Computer use + TV/video watching + video games

^c^ The total MVPA in and outside of school

^d^ 0–100 scale, per 10 units

## Discussion

The present survey provided revised data regarding physical activity levels and sedentary behaviors among middle school students in Beijing. Our results showed that approximately one-third of the students reported MVPA involvement during school for ≥1 h/day. As was previously mentioned, national guidelines require that there must be a daily, 60-minute, after-class exercise period in all schools [23]. Our data suggests that the majority of sampled students did not meet the guidelines.

According to a report of the 2003 Global School-based Student Health Survey, only 21% of Chinese students living in Beijing between the ages of 13 and 15 are engaging in physical activities for at least 60 minutes per day outside of physical education classes [8]. Approximately 34.9% of our surveyed students reported engaging in MVPA outside school for ≥1 h/day. Although these studies differed in terms of sample characteristics, survey periods, and other potential factors, it is possible that student involvement in physical activity has increased during the past decade. However, as the study was conducted only in Beijing and considered relatively few schools, this may not provide conclusive evidence for an increase in physical activity in Chinese students.

Accumulated evidence showed that higher levels of sedentary behavior were associated with higher risk of obesity, less healthful diet, and lower cardiorespiratory fitness in children and adolescents [27,28,29]. Consistent with previous reports [6, 7, 30], we found a high level of academic study-related sedentary behavior such as reading, writing, and drawing. It is said that Chinese students are under pressure to perform well in academic settings [7]. The European Youth Heart Study showed that prolonged screen time during leisure time in adolescence are associated with unfavorable levels of several cardiovascular risk factors in young adulthood. A recent study reported that 26.1% of adolescents were exposed to screen time for ≥2 h/day in China [31]. TV viewing and playing on the computer were found to be the most prevalent screen time behaviors among Chinese adolescents [32]. In our current study, approximately 42.9% of the students reported screen time for ≥2 h/day. The high prevalence of prolonged screen time in our study was mainly attributed to the computer use. Approximately 22.7% students reported using the computer for ≥2 h/day; this usage was previously reported to be 5.4% [33]. Studies from other countries also showed that, during the last decade, time allocated to TV viewing has decreased but has been replaced by an increase in PC use among adolescents [34,35,36]. With the increase in ownership of computers, screen time may continue to increase among Chinese students [9]. Thus exploration of the factors associated with prolonged screen time is critical for preventive purposes.

In concordance with the results of other studies [4,12], our study found boys reported more hours both of physical activity and sedentary activity (computer use, TV/video watching and games) than girls. Girls took part in more study related activities, as reading/writing/drawing, as found in previous studies [9, 37]. In the review by Van Der Horst and his colleagues [38], boys were also found to be positively associated with physical activity and sedentary behavior. It is concluded that variables that are consistently associated with physical activity, do not always have the opposite association with sedentary behavior [38]. Physical activity and sedentary behavior have their own determinants [39,40].

Student attitudes toward physical education have been associated with physical activity participation [13,14]. Our study also found that higher attitudes toward physical education were associated with an increased odds of meeting the physical activity guidelines. As mentioned before, middle and high schools are required to provide at least two physical education classes per week. However, a few studies have explored student attitudes toward physical education in China [24, 41]. Understanding student attitudes toward physical education is important, because it can aid teachers in improving teaching content and forming positive attitudes toward physical education among students [42], which in turn may promote physical activity among students.

Mixed results have been reported for studies on urban, suburban, and rural differences in physical activity in students. It was reported rural adolescents were less physically active than urban youth in the USA and Iceland [14, 43]. However, the opposite trend was also noted by other researchers [17,18]. In this study, we found that students in suburban schools reported less involvement in physical activity than students in urban schools did. The difference may partly be due to the lack of access to sports facilities that students in suburban schools experience, compared with students in urban areas. In China, urban areas usually have higher levels of socioeconomic status (SES) compared to suburban/rural areas. A recent study showed that lower SES home environments provided fewer opportunities for physical activity [44]. Due to the lack of individual data on socioeconomic status in this study, further interpretation of the data on urban and suburban differences in physical activity is limited. A further study detailing the physical activity opportunities, resources, environment, and socioeconomic status between urban and suburban schools should be carried out.

### Limitations

Several limitations of this study must be considered. First, the cross-sectional data limits the inference of causality in the increased prevalence of MVPA outside of school. Second, data were acquired from a small sample of schools in Beijing; thus, the findings may not be easily generalized to the population from which the sample was drawn, and it may not accurately represent the national population. Third, data were obtained through a self-report questionnaire; therefore, reporting bias (over-reporting "physical activity" or under-reporting "sedentary behavior") could have influenced the results [45]. Last, we did not have individual-level data on socioeconomic status for our study sample, so we cannot future interpret the disparities in physical activity between urban and suburban areas in this study.

## Conclusion

In summary, the majority of our students did not meet the current physical activity recommendations. We observed that about half of the students spent excessive time engaging in sedentary behaviors, such as academic related-activities and screen-based activities. Findings from this study highlight a positive association between student attitudes toward physical education and physical activity. Studies are needed to further explore the role of student attitudes toward physical education in promoting physical activity among Chinese students.

## Supporting Information

S1 Appendix Questionnaire. (DOCX)

S1 Dataset Detailed data of this paper. (XLS)
