# Supplementary material for: Study on Current Levels of Physical Activity and Sedentary Behavior among Middle School Students in Beijing, China
Source: PLoS One. 2015 Jul 16;10(7):e0133544. doi: 10.1371/journal.pone.0133544 (PMC4504466; doi:10.1371/journal.pone.0133544)
Supplement: S1 Appendix — (DOCX) [file pone.0133544.s001.docx]

**Questionnaire**

**一 Background demographics**

| 1 age：□□ years |
| --- |
| 2sex：1 male 2 female □ |
| 3 Grade：1 first grade 2 second grade 3 third grade □ |
| 3 During the school semester, do you live away from home in or near school? □  0 no  1 yes |
| 4 Do you go home for each the weekend? □  0 no  1 yes |
|  |

**二 home activities**

| Activity type | During the past week, did you do this chore? | How much time did you spend per day, on average? |
| --- | --- | --- |
| Buy food for your household | **0** no **1** yes □ | □□□ |
| Prepare and cook food for your household | **0** no **1** yes □ | □□□ |
| Wash and iron clothes | **0** no **1** yes □ | □□□ |
| Clean the house | **0** no **1** yes □ | □□□ |

**三 smoking**

1 Have you ever smoked cigarettes (including hand-rolled or device-rolled)？ □

0 never smoked

1 yes

2 How old were you when you started to smoke? □□

3 Do you still smoke cigarettes now?□

0 no

1 yes

4 How many cigarettes do you smoke per day? □□

5 How long ago did you stop smoking? (months) □□

**四 ALCOHOL CONSUMPTION**

1 did you drink beer or any other alcoholic beverage?□

0 no （skip to the next section）

1yes

2 How often did you drink beer or any alcoholic beverage?□

1 almost every day

2 3-4 times a week

3 once or twice a week

4 once or twice a month

5 no more than once a month

**五** SOFT DRINK AND SUGARED FRUIT DRINK CONSUMPTION

1 did you drink soft drinks or sugared fruit drinks?□

0 no （kip to the next section）

1 yes

2 How often did you drink soft drinks or sugared fruit drinks?□

1 almost every day

2 3-4 times a week

3 once or twice a month

4 once or twice a month

5 no more than once a month

**六** PHYSICAL ACTIVITIES

1How many hours each day do you usually sleep, including daytime and nighttime? （hours） □□.□

2 Do you participate in any physical exercises before or after school or on the weekend, including relatively intense physical exercises, such as volleyball, soccer, badminton, and long distance running? □

0 no

1 yes

3 How many times do you participate in any physical exercises before or after school or on the weekend each week? □□

4 On average, for how long do you participate in these physical exercises each？ （minutes）□□□

Physical Activities： Activities Before or After School or on the Weekend

| Activity type | Do you participate in this activity before or after school or on the weekend? | How much time do you spend during a typical day?  Mon ----- Fri Weekend | |
| --- | --- | --- | --- |
| Martial arts (Kung Fu, etc.) | **0** no **1** yes □ | □□□ | □□□ |
| Gymnastics, dancing, acrobatics | **0** no **1** yes □ | □□□ | □□□ |
| Track and field (running, etc.), swimming | **0** no **1** yes □ | □□□ | □□□ |
| Soccer, basketball, tennis | **0** no **1** yes □ | □□□ | □□□ |
| Badminton, volleyball | **0** no **1** yes □ | □□□ | □□□ |
| Other (ping pong, Tai Chi, etc.) | **0** no **1** yes □ | □□□ | □□□ |

**Sedentary Activities**: Activities Before or After School or on the Weekend

| Activity type | Do you participate in this activity before or after school or on the weekend? | How much time do you spend during a typical day?  Mon ----- Fri Weekend | |
| --- | --- | --- | --- |
| TV | **0** no **1** yes □ | □□□ | □□□ |
| Videotapes, VCDs, DVDs | **0** no **1** yes □ | □□□ | □□□ |
| Watching movies and videos online | **0** no **1** yes □ | □□□ | □□□ |
| Video games | **0** no **1** yes □ | □□□ | □□□ |
| Surfing the internet | **0** no **1** yes □ | □□□ | □□□ |
| Participating in chat rooms | **0** no **1** yes □ | □□□ | □□□ |
| Playing computer games, etc. | **0** no **1** yes □ | □□□ | □□□ |
| Doing homework | **0** no **1** yes □ | □□□ | □□□ |
| Extracurricular reading (books, newspapers and magazines), writing, drawing | **0** no **1** yes □ | □□□ | □□□ |
| Toy cars, puppets, board games | **0** no **1** yes □ | □□□ | □□□ |

5 How many times do you participate in physical exercises class in school (in class or at recess) each week? □

6 Do you have any other physical exercise in school (at recess, PE except? □

0 no

1 yes

**Activities in School**

| Activity type | Do you participate in this activity in school? | How much time do you spend each week?  Monday ----- Friday |
| --- | --- | --- |
| Martial arts (Kung Fu, etc.) | **0** no **1** yes □ | □□□ |
| Gymnastics, dancing, acrobatics | **0** no **1** yes □ | □□□ |
| Track and field (running, etc.), swimming | **0** no **1** yes □ | □□□ |
| Soccer, basketball, tennis | **0** no **1** yes □ | □□□ |
| Badminton, volleyball | **0** no **1** yes □ | □□□ |
| Other (ping pong, Tai Chi, etc.) | **0** no **1** yes □ | □□□ |

**七 Diet Knowledge**

Do you know about the Dietary Guidelines for Chinese Residents?

0 yes

1 no

**Diet Knowledge**

| Statement  Please use 1-5 to describe if you strongly disagree, somewhat disagree, neutral, somewhat agree, or strongly agree with this statement.  * Please note that the question is not asking about your actual habits. | 1 strongly disagree  2 disagree  3 neutral  4 agree  5 strongly agree  9 unknown |
| --- | --- |
| Choosing a diet with a lot of fresh fruits and vegetables is good for one’s health. | □**U377a** |
| Eating a lot of sugar is good for one’s health. | □**U378a** |
| Eating a variety of foods is good for one’s health. | □**U379a** |
| Choosing a diet high in fat is good for one’s health. | □**U380a** |
| Choosing a diet with a lot of staple foods [rice and rice products and wheat and wheat products] is not good for one’s health. | □**U381a** |
| Consuming a lot of animal products daily (fish, poultry, eggs and lean meat) is good for one’s health. | □**U382**a |
| Reducing the amount of fatty meat and animal fat in the diet is good for one’s health. | □**U383a** |
| Consuming milk and dairy products is good for one’s health. | □**U384a** |
| Consuming beans and bean products is good for one’s health. | □**U385a** |
| Physical activities are good for one’s health. | □**U386a** |
| Sweaty sports or other intense physical activities are not good for one’s health. | □**U387a** |
| The heavier one’s body is, the healthier he or she is. | □**U388a** |

**Food Preferences**

| Food item  Please use 1-5 to describe how much you like this food: dislike very much, dislike, neutral, like, or like very much. | 1 dislike very much  2 dislike  3 neutral  4 like  5 like very much  9 does not eat this food |
| --- | --- |
| Fast food (KFC, pizza, hamburgers, etc.) | □**U389a** |
| Salty snack foods (potato chips, pretzels, French fries, etc.) | □**U390a** |
| Fruits | □**U391a** |
| Vegetables | □**U392a** |
| Soft drinks and sugared fruit drinks | □**U393a** |

**Activity Preferences**

| Activity type  Please use 1-5 to describe how much you like to participate in this activity: dislike very much, dislike, neutral, like, or like very much.  * Please note we are asking if you participate in the activity, not just watch the activity or games on TV or as a spectator attending an event. | 1 dislike very much  2 dislike  3 neutral  4 like  5 like very much  9 does not participate |
| --- | --- |
| Walking, Tai Chi | □**U394a** |
| Sports (ping pong, badminton, tennis, soccer, basketball, volleyball) | □**U395a** |
| Body building | □**U396a** |
| Watching TV | □**U397a** |
| Playing computer/video games, surfing the internet | □**U398a** |
| Reading | □**U399a** |

**八** Students’ Attitudes Toward Physical Education

| 5 = Strongly agree 1 = Strongly disagree | 1-------------5 |
| --- | --- |
| 1 The games I learn in physical education make my physical education class interesting for me. | □ |
| 2 The games I learn in my physical education class make learning unpleasant for me. |  |
| 3 The games I learn in my physical education class get me excited about physical education. |  |
| 4 My physical education teacher makes my physical education class seem unimportant to me. |  |
| 5 I feel the games I learn in physical education make my physical education class boring for me. |  |
| 6 I feel the games I learn in my physical education class are useless to me. |  |
| 7 The games I learn in my physical education class seem important to me. |  |
| 8 My physical education teacher makes my physical education class seem important to me. |  |
| 9 My physical education teacher makes my physical education class interesting for me. |  |
| 10 The games I learn in my physical education class are useful to me. |  |
| 11 I feel my physical education teacher makes learning in my physical education class fun for me. |  |
| 12 I feel my physical education teacher makes my physical education class boring for me. |  |
| 13 I feel the games I learn in my physical education class are valuable to me. |  |
| 14 The games I learn in my physical education class seem unimportant to me. |  |
| 15 My physical education teacher makes learning in my physical education class unpleasant for me. |  |
| 16My physical education teacher makes my physical education class useful for me. |  |
| 17I feel my physical education teacher makes learning in my physical education class valuable for me. |  |
| 18I feel my physical education teacher makes learning in my physical education class useless for me. |  |
| 19My physical education teacher gets me excited about physical education. |  |
| 20 I feel the games I learn in my physical education class make learning fun for me. |  |
